# Influence of anti–tumor necrosis factor therapy on cancer incidence in patients with rheumatoid arthritis who have had a prior malignancy: Results from the British Society for rheumatology biologics register

**DOI:** 10.1002/acr.20129

**Published:** 2010-06

**Authors:** W G Dixon, K D Watson, M Lunt, L K Mercer, K L Hyrich, D P M Symmons, British Society For Rheumatology Biologics Register Control Centre Consortium, British Society for Rheumatology Biologics Register

**Affiliations:** The University of ManchesterManchester, UK

## Abstract

**Objective:**

To explore the influence of anti–tumor necrosis factor (anti-TNF) therapy upon the incidence of cancer in patients with rheumatoid arthritis (RA) and prior malignancy.

**Methods:**

Using data from the British Society for Rheumatology Biologics Register, a national prospective observational study established in 2001, we identified 293 patients with a prior malignancy from over 14,000 patients with RA. We compared rates of incident malignancy in 177 anti-TNF–treated patients and 117 patients with active RA treated with traditional disease-modifying antirheumatic drugs (DMARDs), all with prior malignancy. One patient switched therapy and contributed to both cohorts.

**Results:**

The rates of incident malignancy were 25.3 events/1,000 person-years in the anti-TNF cohort and 38.3/1,000 person-years in the DMARD cohort, generating an age- and sex-adjusted incidence rate ratio of 0.58 (95% confidence interval 0.23–1.43) for the anti-TNF–treated cohort compared with the DMARD cohort. Of the patients with prior melanomas, 3 (18%) of 17 in the anti-TNF cohort developed an incident malignancy, compared with 0 of 10 in the DMARD cohort.

**Conclusion:**

The way in which UK rheumatologists are selecting patients with RA and prior malignancy to receive anti-TNF therapy is not leading to an increased risk of incident malignancy. Although reassuring, these results should not be interpreted as indicating that it is safe to treat all RA patients with prior malignancy with anti-TNF therapy.

## INTRODUCTION

Tumor necrosis factor (TNF) is a pivotal cytokine in the inflammatory synovium of patients with rheumatoid arthritis (RA). This discovery led to the successful development of anti-TNF therapy, thereby significantly advancing drug treatment for patients with RA. Although important in the pathophysiology of RA, TNF also has many physiologic roles, including host defense and tumor surveillance (1). Therefore, despite good proven efficacy, there have always been concerns about the safety of anti-TNF therapy.

The exact relationship between TNF and cancer is unclear. At high doses, TNF has been used as a treatment for some malignancies, including melanoma and sarcoma (2). Conversely, anti-TNF therapy has been suggested as a possible treatment for cancer-associated cachexia (3,4). It is plausible that TNF has different roles at different concentrations during different stages of tumor genesis. Similarly, anti-TNF therapy may have a spectrum of influence upon de novo malignancy, carcinoma in situ (CIS), and preexisting malignancy.

The hypothetical increased risk of new or recurrent malignancy in patients with prior malignancy led researchers to exclude such patients from randomized clinical trials of anti-TNF therapy for RA (5–7). Clinical trial data and open-label extension studies are thus limited in their ability to address the question of whether anti-TNF therapy influences the malignancy rate in patients with prior cancer. Observational studies are similarly limited by the usual clinical practice, often influenced by local or national guidelines (8,9). In the UK, the British Society for Rheumatology (BSR) guidelines for anti-TNF prescription state that “caution should be exercised in the use of anti-TNF therapies in patients with previous malignancy. The potential benefits of treatment need to be considered against the risks related to potential recurrence of the specific malignancy. If patients have been free of any recurrence of their malignancy for 10 years there is no evidence for a contraindication to anti-TNF therapy” (8). The potential harm of anti-TNF therapy in patients with RA with prior malignancy has never been quantified, making an evidence-based calculation of the benefit/harm balance impossible. The aim of this study, therefore, was to explore the influence of anti-TNF therapy on the incidence of cancer in patients with RA and prior malignancy.

## PATIENTS AND METHODS

The subjects for this analysis were participating in a large national prospective observational study, the BSR Biologics Register (BSRBR). The methods have been described in detail elsewhere (10). In brief, the study was established in 2001 in order to examine the long-term safety of biologic drugs. UK national guidelines recommended that anti-TNF drugs should be reserved for patients with active RA (Disease Activity Score in 28 joints [DAS28] >5.1) despite previous therapy with at least 2 disease-modifying antirheumatic drugs (DMARDs), and that “any clinician prescribing these medications must (with the patient's permission) undertake to register the patient with the [BSRBR] and forward information on dosage, outcome and toxicity on a six-monthly basis” (11). Recruitment targets of 4,000 patients for the 3 anti-TNF drugs etanercept, infliximab, and adalimumab were met in 2005, 2007, and 2008, respectively. No accurate figures for anti-TNF penetration in the UK RA population exist, although estimates of ∼7% have been suggested (12). Before recruitment targets were met, we estimated that >80% of anti-TNF–treated patients with RA in the UK were registered with the BSRBR. Ethical approval for this study was obtained in December 2000 from the Multicentre Research Ethics Committee for the Northwest of England.

### Anti-TNF cohort

Analysis was restricted to patients registered with the BSRBR with a physician diagnosis of RA who were commencing an anti-TNF drug as their first biologic drug. Patients registered >6 months after the start of biologic agent therapy were excluded. All patients in this study were registered prior to September 30, 2007.

### Comparison cohort

A cohort of patients who had never taken biologic agents and had active RA was being recruited in parallel (Appendix A) and followed with identical methodology (10). These patients had a physician diagnosis of RA with active disease (guideline DAS28 ≥4.2) despite current treatment with a traditional DMARD, and had never taken biologic agents. Comparison patients also had to be registered prior to September 30, 2007.

### Identification of prior malignancy

Analysis was limited to patients with prior malignancy. At registration, all patients were linked to the UK National Health Service Information Centre (NHS IC; formerly the Office for National Statistics), which collates data from the 8 regional English cancer registries, in addition to similar registers in Wales, Scotland, and Northern Ireland. This provided details of all historic cancers, including date of diagnosis, type, and anatomic site. Prior malignancies were defined as malignancies identified by the NHS IC with a diagnosis date prior to the first dose of anti-TNF therapy, or prior to the registration date for the comparison cohort. CIS and nonmelanoma skin cancer were excluded. Where patients had >1 prior malignancy, the most recent malignancy was reported.

### Baseline assessment

Baseline information for both cohorts included demographics, disease duration, 28 swollen and 28 tender joint counts, erythrocyte sedimentation rate and/or C-reactive protein level, and patient global assessment, which enables calculation of a DAS28 score (13). Patients completed a Health Assessment Questionnaire (HAQ) adapted for British use (14). Details of all previous and current DMARD therapy and all other current medication were obtained, as well as smoking history and comorbidity.

### Followup

Data on the occurrence of adverse events were captured in 3 ways: 6-monthly rheumatologist questionnaire, 6-monthly patient diary, and by flagging with the NHS IC, who provided information on incident malignancy and mortality, including cause of death (coded according to the International Classification of Diseases, Tenth Revision). It has been estimated that the NHS IC accurately captures ∼90% of incident cancers (15). After 3 years of followup, consultant questionnaires were sent annually, and patient diaries were no longer sent.

### Definition of incident malignancy

Incident malignancies were defined as malignancies diagnosed after the first dose of anti-TNF therapy for the anti-TNF cohort, or after the registration date for the DMARD cohort. New primaries, local recurrence, and metastases were all included as incident cancers. CIS and nonmelanoma skin cancer were excluded, as were benign cancers and malignancies known to be present on the date of registration.

### Verification of incident malignancy

If cancers were reported from any source, clinicians were asked to provide further information for these events. Cancers were categorized as definite if there was histologic confirmation or if they were reported by the NHS IC. Cancers were categorized as probable if the patients received definite anticancer treatment such as chemotherapy or radiotherapy, if there was diagnostic imaging, if there was direct visualization of the tumor, or if the malignancy was reported on the death certificate. Other cancers were labeled as possible if they were consultant reported and did not fulfill the above criteria. Cancers reported by the patient without any further verification from the rheumatologist or NHS IC were excluded. All supporting information was independently reviewed by 2 clinicians (WGD and LKM). Any disagreement about verification category was resolved by discussion.

### Statistical analysis

Followup time was calculated from the date of the first anti-TNF drug use for the anti-TNF–treated cohort, or from the registration date for the comparison cohort, to September 30, 2007 or the death date, whichever occurred first. Malignancy data were captured from all 3 sources until April 2008 to allow for delays in notification of incident malignancies to the BSRBR. Within the anti-TNF cohort, patients could switch between anti-TNF drugs. The anti-TNF cohort contributed person-years of followup even if the anti-TNF therapy was stopped. Malignancies were attributed to anti-TNF therapy irrespective of drug discontinuation. In other words, malignancies occurring both during active anti-TNF therapy and after stopping anti-TNF therapy were attributed to the anti-TNF cohort. Patients ever treated with a non–anti-TNF biologic drug (e.g., anakinra or rituximab) were excluded completely from the analysis. Patients initially registered in the comparison cohort who subsequently received an anti-TNF drug contributed person-years to the comparison cohort up to the date that the anti-TNF drug was started, and contributed subsequent followup to the anti-TNF cohort.

Patients could develop a second or subsequent malignancy following the date of their first incident malignancy. Followup was therefore not censored at the time of incident cancer diagnosis. However, a sensitivity analysis was conducted, excluding time and events after the first malignancy. The influence of time since prior malignancy was examined by stratifying the data according to time since prior cancer (more or less than 10 years).

Malignancy rates are presented as events/1,000 person-years with 95% confidence intervals (95% CIs). Incidence rate ratios (IRRs) were calculated using Cox regression, comparing between the anti-TNF cohort and the DMARD cohort. Adjustment was made for age and sex. A propensity score was calculated based on age, sex, disease duration, baseline DAS28 and HAQ scores, year of entry, and smoking status. Propensity-adjusted estimates were calculated by stratifying the propensity score into deciles. Multiple imputation was used to avoid bias caused by missing data (there were 3 subjects with missing disease duration, 5 with missing DAS28, and 32 with missing HAQ score). Nelson-Aalen cumulative incidence plots were generated to explore time-dependent incidence in the two cohorts. All analysis was done using Stata, versions 9.2 and 10.1 (Stata).

## RESULTS

**Figure 1:**
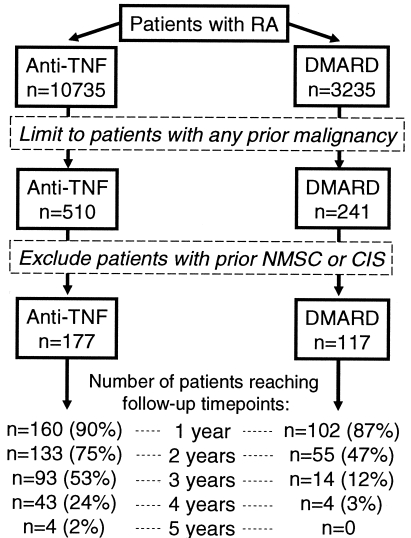
Flow chart of patients in study. RA = rheumatoid arthritis; anti-TNF = anti–tumor necrosis factor; DMARD = disease-modifying antirheumatic drug; NMSC = nonmelanoma skin cancer; CIS = carcinoma in situ.

Of the patients, 10,735 received anti-TNF therapy and no other biologic agent therapy. The initial anti-TNF drug was etanercept for 3,971 patients, infliximab for 3,352 patients, and adalimumab for 3,412 patients. The comparison cohort consisted of 3,235 DMARD-treated patients. After linkage with the NHS IC, 293 patients with a prior history of malignancy (excluding CIS and nonmelanoma skin cancer) were identified, 177 (1.6%) of 10,735 in the anti-TNF cohort and 117 (3.6%) of 3,235 in the comparison cohort (Figure 1). One patient who originally registered in the DMARD cohort started anti-TNF therapy and therefore contributed person-years to both cohorts. All subsequent analyses are restricted to these patients. Of the 177 patients in the anti-TNF cohort, 46 received >1 anti-TNF drug.

The baseline characteristics are shown in Table 1. The anti-TNF cohort was younger, comprised of proportionally more women, and, as expected, had more severe disease than the comparison cohort. The anti-TNF cohort was more likely to be receiving steroids at baseline, and included fewer current or former smokers. Subtypes of prior malignancy were balanced in the two cohorts. Approximately 80% of the prior malignancies were solid tumors, with the remainder approximately divided into lymphoproliferative malignancies and melanomas. Proportionally more prior malignancies were diagnosed >10 years before registration for the anti-TNF cohort (58%) compared with the DMARD cohort (39%).

**Table 1:** Baseline characteristics*

	DMARD (n = 117)	All anti-TNF (n = 177)	*P*
Age, mean ± SD years	66 ± 10	62 ± 10	0.002
Women, %	74	81	0.110
DAS28, mean ± SD	5.0 ± 1.3	6.7 ± 1.2	0.0001
HAQ score, mean ± SD	1.6 ± 0.7	2.2 ± 0.5	0.0001
Disease duration, median (IQR) years	9 (2–18)	11 (6–18)	0.0083
Prior DMARDs, median (IQR)	2 (1–4)	4 (3–5)	0.0001
Baseline steroid use	39 (33)	90 (51)	0.003
Smoking			
Current	25 (21)	32 (18)	0.011
Former	61 (52)	67 (38)	
Never	31 (27)	77 (44)	
Entry year			
Pre-2003	0	15 (8)	< 0.0001
2003	6 (5)	57 (32)	
2004	27 (23)	49 (28)	
2005	41 (35)	28 (16)	
2006 or after	43 (37)	28 (16)	
Prior malignancy			
Solid	96 (82)	147 (83)	0.795
Lymphoproliferative	11 (9)	13 (7)	
Melanoma	10 (8)	17 (10)	
Time from most recent prior malignancy to registration			
Median (IQR) years	8.5 (4.7–14.1)	11.5 (5.8–17.1)	0.027
>10 years preregistration	46 (39)	102 (58)	0.002

* Values are the number (percentage) unless otherwise indicated. DMARD = disease-modifying antirheumatic drug; anti-TNF = anti–tumor necrosis factor; DAS28 = Disease Activity Score in 28 joints; HAQ = Health Assessment Questionnaire; IQR = interquartile range.

The total followup time was 750 person-years: 515 for the anti-TNF cohort and 235 for the DMARD cohort. Patients in the anti-TNF cohort contributed a median followup time of 3.1 person-years, compared with 1.9 person-years for patients in the DMARD cohort. Only 12 (4%) of 293 patients had no returned consultant questionnaires during their followup.

**Figure 2:**
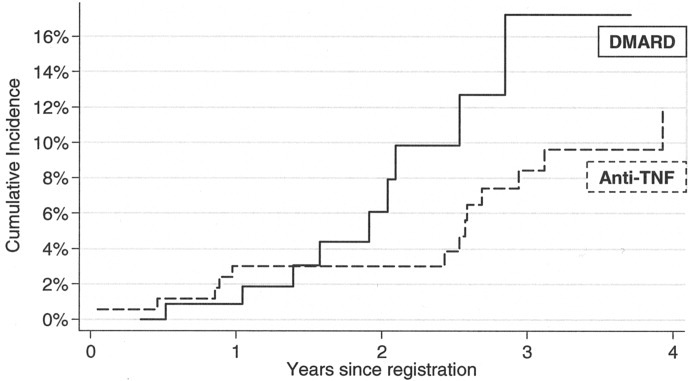
Cumulative incidence of malignancy in patients with prior malignancy by Nelson-Aalen plot. DMARD = disease-modifying antirheumatic drug; anti-TNF = anti–tumor necrosis factor.

There were 13 incident malignancies in 11 patients identified in the anti-TNF cohort compared with 9 malignancies in 9 patients in the DMARD cohort (Table 2 and Figure 2). There were no patient-reported malignancies that were not verified by a consultant. The resultant rates of malignancy were 25.3 events/1,000 person-years in the anti-TNF cohort and 38.3 events/1,000 person-years in the DMARD cohort. The age- and sex-adjusted IRR was 0.58 (95% CI 0.23–1.43) for the anti-TNF–treated patients compared with the DMARD cohort. The propensity-adjusted estimate was 0.45 (95% CI 0.09–2.17). A sensitivity analysis, censoring followup at the first incident malignancy, resulted in an age- and sex-adjusted IRR of 0.52 (95% CI 0.21–1.33) and a propensity-adjusted IRR of 0.47 (95% CI 0.10–2.22) (Table 3). Stratifying by time since prior malignancy did not reveal any effect measure modification. The age- and sex- adjusted IRR was 0.71 (95% CI 0.18–2.79) for malignancies that occurred <10 years prior to registration, and was 0.63 (95% CI 0.10–4.11) for malignancies >10 years prior to registration.

**Table 2:** Rate of incident cancers in patients with prior malignancy*

	DMARD (n = 117)	All anti-TNF (n = 177)
Person-years of followup	235	515
Person-years of followup per patient, median (IQR)	1.9 (1.3–2.7)	3.1 (2.0–3.9)
Incident malignancies, no.	9	13
Patients with incident malignancies, no.	9	11
Rate of incident malignancy/1,000 person-years (95% CI)	38.3 (17.5–72.7)	25.3 (13.4–43.2)
IRR (95% CI)	Referent	0.56 (0.23–1.35)
IRR adjusted for age and sex (95% CI)	Referent	0.58 (0.23–1.43)
Propensity-adjusted IRR (95% CI)	Referent	0.45 (0.09–2.17)

* DMARD = disease-modifying antirheumatic drug; anti-TNF = anti–tumor necrosis factor; IQR = interquartile range; 95% CI = 95% confidence interval; IRR = incidence rate ratio.

**Table 3:** Sensitivity analysis: rate of incident cancers in patients with prior malignancy: followup censored after first incident malignancy*

	DMARD (n = 117)	All anti-TNF (n = 177)
Person-years of followup	232	503
Person-years followup per patient, median (IQR)	1.8 (1.3–2.6)	3.0 (1.9–3.9)
Incident malignancies, no.	9	11
Patients with incident malignancies, no.	9	11
Rate of incident malignancy/1,000 person-years (95% CI)	38.7 (17.7–73.5)	21.9 (10.9–39.2)
IRR (95% CI)	Referent	0.51 (0.2–1.27)
IRR adjusted for age and sex (95% CI)	Referent	0.52 (0.21–1.33)
Propensity-adjusted IRR (95% CI)	Referent	0.47 (0.10–2.22)

* DMARD = disease-modifying antirheumatic drug; anti-TNF = anti–tumor necrosis factor; IQR = interquartile range; 95% CI = 95% confidence interval; IRR = incidence rate ratio.

A clinical table of the timings and anatomic sites of the prior and incident malignancies is shown in Table 4. Of the 6 patients with a malignancy <10 years prior to starting anti-TNF therapy who later developed an incident malignancy, 3 had a prior melanoma. Three (18%) of the 17 patients with prior melanomas in the anti-TNF cohort developed an incident malignancy, compared with 0 of 10 in the DMARD cohort.

**Table 4:** Prior and incident cancers, clinical table*

			Prior cancer		Incident cancer		
Cohort and patient number	Age band, years	Sex	Site of prior cancer	Time preregistration, years	Site of incident cancer	Definite/probable/ possible	Time postregistration, years
Anti-TNF cohort							
1	60–69	Female	Lung	0.5	Spinal and liver metastases	Definite	0.9
2	40–49	Female	Melanoma	2.9	CNS metastases	Probable	0.5
3	50–59	Male	Melanoma	3.5			
First incident cancer					Bladder	Definite	2.4
Second incident cancer					Pleural melanoma	Definite	3.9
4	50–59	Female	Breast	4.6	Neck lump	Probable	2.6
5	60–69	Female	Melanoma	7.5	Multiple metastases (adenocarcinoma)	Definite	3.1
6	50–59	Female	Digestive organ	8.9	Colon	Definite	1.0
7	60–69	Male	Cecum	10.3	Colon	Definite	0.9
8	70–79	Female	Breast	10.9	Colon	Definite	2.6
9	60–69	Female	Kidney	12.8	Kidney with spread to inferior vena cava	Probable	2.5
10	60–69	Female	Breast	15.3			
First incident cancer					Low-grade CLL	Definite	2.7
Second incident cancer					Lung with liver metastases	Definite	2.9
11	70–79	Female	Appendix adenocarcinoma	21	Cholangiocarcinoma	Definite	0.05
DMARD cohort							
12	70–79	Male	Prostate	2.6	Prostate with bony metastases	Probable	1.9
13	50–59	Female	Uterus	4.7	Frontal lobe of brain	Definite	1.0
14	60–69	Female	Breast	5.4	Pancreatic adenocarcinoma with pleural and peritoneal metastases	Definite	0.5
15	60–69	Male	Prepuce	5.9	Penis with metastases	Probable	2.8
16	50–59	Female	Breast	8.6	Anal	Definite	1.6
17	50–59	Female	Thyroid	9.7	Kidney	Definite	1.4
18	70–79	Male	Kidney	9.7	Transitional cell carcinoma of bladder	Definite	2.1
19	60–69	Female	Breast	10.6	Breast with liver and bone metastases	Probable	2.8
20	70–79	Male	Lip	15.2	Lung	Definite	2.0

* Anti-TNF = anti–tumor necrosis factor; CNS = central nervous system; CLL = chronic lymphocytic leukemia; DMARD = disease-modifying antirheumatic drug.

## DISCUSSION

We have shown that in patients with RA and prior malignancy, the rate of incident malignancy is not increased in patients selected to receive anti-TNF therapy after an average of 3 years of followup. The age- and sex-adjusted IRR was 0.58 (95% CI 0.23–1.43) for the anti-TNF–treated cohort compared with the DMARD cohort. This finding is very reassuring at face value. However, it must be interpreted with great care in the context of an observational drug study. We must carefully consider the potential selection factors and biases, and try to understand how they might influence the result.

A major potential bias in this analysis is selection bias. When considering further management for a patient with active RA and prior malignancy, clinicians make a treatment decision based upon available information. They may be more inclined to prescribe anti-TNF therapy if the prior malignancy had a good prognosis. Examples may include prior cancers with a long period in remission, complete surgical excision, or a “low-grade” tumor. In contrast, patients with prior malignancies who had a higher chance of recurrence or metastasis may have had anti-TNF therapy rejected based upon the hypothetical increased risk conferred by the drug. This may lead to an imbalance in the baseline risk of incidence malignancy between the two cohorts, partially explaining the lower rate in the anti-TNF–treated cohort. This hypothesis is supported by the higher proportion of DMARD-treated patients with a prior malignancy <10 years prior to the registration date. Although the broad subtypes of prior malignancy were balanced in the two cohorts, we do not have detailed information about these prior cancers. Another possible imbalance was differential cancer screening prior to study entry between the cohorts treated with anti-TNF therapy and DMARDs. Unfortunately, no information was available on the type or extent of screening in the two cohorts. We cannot know, therefore, whether the likelihood of recurrence differed between the two groups according to the stage, severity, or histology of the original malignancies. Nonetheless, this is the only study setting that can feasibly address this clinically important question. Randomized controlled trials are too small, too short, and exclude the patients of interest. Spontaneous pharmacovigilance studies provide an inaccurate numerator, no denominator, and no comparison cohort.

Following registration, the method of followup was identical for the two cohorts. Any diagnosed incident malignancies would have been reported to the BSRBR with equal likelihood in the two cohorts. There remains the possibility of surveillance bias, in which patients in the anti-TNF cohort have more frequent followup appointments and/or closer clinical scrutiny of new symptoms that might then be diagnosed as an incident malignancy. Such a bias, however, would elevate the rate in the anti-TNF cohort and does not explain our findings.

The differential duration of followup may have an impact on our results. Patients in the anti-TNF–treated cohort were followed up for just over 3 years compared with nearly 2 years for the DMARD cohort. This differential followup time is important only if the rate of cancer changes with time. The cumulative incidence plot in Figure 2 suggests that the rate may accelerate with increasing duration of followup: a possible explanation being a preregistration screen for malignancy that wears off with increasing followup. However, if this increasing rate of malignancy with time is true, extending followup in the DMARD cohort would further increase the rate of incident malignancy in this cohort. Once again, this potential bias does not explain our findings of a lower rate in the anti-TNF–treated cohort. Although it is important to consider time-dependent risk, the number of events was too low to generate any meaningful sensitivity analysis by time bands.

One of the strengths of the BSRBR is its size. It is a national prospective observational study that includes >14,000 patients with RA. Despite this, there were only 293 patients with prior cancer. Only 20 patients developed an incident cancer, limiting the power of the study. The point estimate for the IRR of 0.58 (95% CI 0.23–1.43) has wide 95% CIs, and we cannot be certain of an absence of an increased risk. However, this finding is our current best estimate to address an important clinical question. Despite the wide 95% CIs, this study adds useful information to an area with no prior evidence base.

The reported IRR was adjusted only for age and sex. The low number of incident malignancies limits further adjustment for potential confounders, following the statistical rule that no more than 1 variable should be included in the model per 10 events (16). We must therefore consider whether our results might be explained by residual or unmeasured confounding. The addition of calendar year to the analysis did not change the point estimate. High disease severity is associated with certain malignancies such as lymphoma (17). The imbalance of disease severity at baseline therefore might be expected to increase the malignancy rate in the anti-TNF–treated cohort, which would not explain our findings. The higher proportion of patients who had ever smoked in the DMARD cohort may go some way to explain their higher malignancy rate. Other variables such as steroid use were also imbalanced. Although it is difficult to statistically adjust for these potential confounders, it seems unlikely that residual confounding would explain the magnitude of differential risk in the two cohorts. This is supported by the propensity-adjusted analyses not changing the results. We have previously shown socioeconomic status (SES) in anti-TNF–treated patients to closely reflect the general population distribution (18). The lack of association between SES and anti-TNF exposure means that SES cannot be a confounder.

One way of tackling multiple potential confounders and a low number of outcomes is to use a propensity score. We undertook such an analysis, and adjusting for the propensity score made very little difference to the result.

Prior malignancies were identified using linkage with the NHS IC. It is possible that some patients with prior malignancy were not identified due to an administrative delay from clinical diagnosis to notification from the NHS IC to the BSRBR, and therefore were not included in this analysis. However, such patients should not be systematically different from those included in the study and would not affect the validity of the findings.

Previous reports from the BSRBR have limited analysis to periods with returned consultant followup (19). For this analysis, by using an “ever had drug” model of analysis, followup time beyond the last returned consultant followup questionnaire was also included because malignancies could still be captured robustly from patient diaries and the NHS IC. We continued data capture for 6 months beyond the study cutoff date to maximize data return from all sources.

All incident malignancies were attributed to anti-TNF in patients in the anti-TNF cohort whether or not they continued the drug. Other options would be to restrict analysis to malignancies that were diagnosed while the patient was either actively receiving the drug, or within a lag window of risk beyond the stop date (19). We elected to use an “ever had drug” model because, if there is an association between anti-TNF therapy and malignancy, the clinical latency is not known.

All incident malignancies were consultant reported. Of the 22 reported cancers, 16 were defined as definite and 6 as probable. None were defined as possible. These results are very reassuring. Despite not all malignancies being defined as definite, there was strong supporting evidence for all incident malignancies in the analysis.

It is possible that malignancies identified early in the course of followup may have been preexisting but as yet undiagnosed. Only one incident malignancy was diagnosed in the first 6 months of followup (patient #11). Exclusion of this event from the anti-TNF cohort would further reduce the IRR, supporting the absence of an increased risk with anti-TNF therapy.

Two patients had >1 incident malignancy. Patient #3 had a melanoma 3.5 years prior to anti-TNF therapy, followed by bladder cancer 2.4 years after starting therapy. If followup was censored here, we would miss the pleural melanoma diagnosed after 3.9 years. This may represent a recurrence of the original primary, and is therefore an important case. We did perform a sensitivity analysis excluding time after the first malignancy, which did not significantly alter our point estimate. Only one of the patients who developed an incident malignancy had >1 prior cancer. Patient 12 in the DMARD cohort had both non-Hodgkin's lymphoma and bladder cancer prior to registration.

An interesting question to address would have been the relative likelihood of definite recurrence or metastasis of the original primary cancer. Unfortunately, despite stringent efforts to collect as much information about both the prior and incident malignancies, definite links could not often be made due to a lack of histologic information. For instance, it is possible that the cerebral metastases in patient 2 were either related or not related to the prior melanoma. Because no postmortem was conducted, this information was not available. Any such analysis would therefore be subject to a significant degree of uncertainty.

No previous published studies have been able to address the question as to whether anti-TNF therapy influences the rate of malignancy in patients with prior malignancy. Studies have been undertaken to understand whether anti-TNF therapy increases the rate of de novo malignancy. The results of such studies have ranged from no increased risk in observational studies to a >3-fold increased risk in meta-analyses of randomized controlled trials. These findings have been reviewed elegantly elsewhere (20). Our suggestion that patients with prior melanoma may be at particular risk of incident malignancy fits with the existing literature. Supraphysiologic doses of TNF have been used to treat melanomas (2), suggesting that anti-TNF therapy may be disadvantageous. An observational study from the National Data Bank for Rheumatic Diseases also found an increased risk of melanoma in patients treated with anti-TNF therapy, although the overall risk of malignancy was not increased (21). This further supports a possible melanoma-specific risk.

In summary, we have shown that the way in which UK rheumatologists are selecting their patients with RA and prior malignancy to receive anti-TNF therapy is not leading to an increased risk of incident malignancy over the period of followup studied. The results should not be interpreted as indicating that it is safe to treat all RA patients with prior malignancy with anti-TNF therapy.

## AUTHOR CONTRIBUTIONS

All authors were involved in drafting the article or revising it critically for important intellectual content, and all authors approved the final version to be submitted for publication. Dr. Symmons had full access to all of the data in the study and takes responsibility for the integrity of the data and the accuracy of the data analysis.

**Study conception and design.** Dixon, Watson, Mercer, Hyrich, Symmons.

**Acquisition of data.** Dixon, Watson, Hyrich.

**Analysis and interpretation of data.** Dixon, Watson, Lunt, Mercer, Hyrich, Symmons.

## ROLE OF THE STUDY SPONSOR

The BSR commissioned the BSRBR as a UK national project to investigate the safety of biologic agents in routine medical practice. DPMS and KLH are principal investigators on the BSRBR. The BSR receives restricted income from UK pharmaceutical companies, presently Abbott Laboratories, Biovitrum, Roche, Schering Plough, and Wyeth Pharmaceuticals. This income finances a wholly separate contract between the BSR and the University of Manchester, who provide and run the BSRBR data collection, management, and analysis services. The principal investigators and their team have full academic freedom and are able to work independently of pharmaceutical industry influence. All decisions concerning analyses, interpretation, and publication are made autonomously of any industrial contribution. Members of the Manchester team, BSR trustees, committee members, and staff complete an annual declaration in relation to conflicts of interest.

## APPENDIX A

### MEMBERS OF THE BSRBR CONTROL CENTRE CONSORTIUM

The members of the BSRBR Control Centre Consortium are Nicola Maiden (Antrim Area Hospital, Antrim), Tom Price (Cannock Chase Hospital, Cannock Chase), Neil Hopkinson (Christchurch Hospital, Christchurch), Sheila O'Reilly (Derbyshire Royal Infirmary, Derby), Lesley Hordon (Dewsbury and District Hospital, Dewsbury), Ian Griffiths (Freeman Hospital, Newcastle-upon-Tyne), Duncan Porter (Gartnavel General Hospital, Glasgow), Hilary Capell (Glasgow Royal Infirmary, Glasgow), Andy Hassell (Haywood Hospital, Stoke-on-Trent), Romela Benitha (Hope Hospital, Salford), Ernest Choy (King's College Hospital, London), David Walsh (Kings Mill Centre, Sutton-In Ashfield), Paul Emery (Leeds General Infirmary, Leeds), Susan Knight (Macclesfield District General Hospital, Macclesfield), Ian Bruce (Manchester Royal Infirmary, Manchester), Allister Taggart (Musgrave Park Hospital, Belfast), David Scott (Norfolk and Norwich University Hospital, Norwich), Paul Thompson (Poole General Hospital, Poole), Fiona McCrae (Queen Alexandra Hospital, Portsmouth), Rhian Goodfellow (Royal Glamorgan Hospital, Glamorgan), George Kitas (Russells Hall Hospital, Dudley), Ronald Jubb (Selly Oak Hospital, Selly Oak), Rikki Abernethy (St Helens Hospital, St Helens), Shane Clarke, Sandra Green (Weston General Hospital, Weston-super-Mare), Paul Sanders (Withington Hospital, Manchester), and Amanda Coulson (Withybush General Hospital, Haverfordwest).
